# Epidermodysplasia Verruciformis: A Case Series

**DOI:** 10.7759/cureus.78058

**Published:** 2025-01-27

**Authors:** Priyanka RM, Manuvidhya H, Anuradha Priyadarshini, Leena Dennis Joseph, Adikrishnan Swaminathan

**Affiliations:** 1 Dermatology, Venereology, Leprosy, Sri Ramachandra Institute of Higher Education and Research, Chennai, IND; 2 General Pathology, Sri Ramachandra Institute of Higher Education and Research, Chennai, IND

**Keywords:** edv, epidermodysplasia verruciformis, ev, hpv, lewandowski-lutz dysplasia, squamous cell carcinoma, vulval carcinoma

## Abstract

Epidermodysplasia verruciformis (EV) is an uncommon disease that is inherited autosomal recessively. It has a heightened vulnerability to human papillomavirus (HPV) infection, which manifests as hyperpigmented or hypopigmented macular lesions, lesions resembling pityriasis versicolor (PV) and planar warts, and an increased risk of progressing to skin cancer, especially in sun-exposed areas. We report three cases of EV with varying presentations. One patient, a long-standing case of EV, developed squamous cell carcinoma (SCC) in the vulva and inguinal region. Another patient had a rare association with palmar pits, while the third patient presented with classical EV. All our patients were prescribed oral retinoids and advised to practice stringent photoprotection after histopathology confirmed evidence of EV.

## Introduction

Epidermodysplasia verruciformis (EV) is a rare autosomal recessive genodermatosis. It is characterized by increased susceptibility to human papillomaviruses (HPVs) and typically starts in childhood [1]. Most often, mutations in the transmembrane channel gene EVER1/TMC6 or EVER2/TMC8 genes, which are found on chromosome 17q25, are the cause. EV presents as pityriasis versicolor (PV)-like lesions, plane warts, and verrucous plaques, which typically appear on sun-exposed areas of the face, torso, neck, and extremities [2]. Patients with EV are prone to developing non-melanoma skin cancers, with commonly associated dysplastic and malignant changes, including actinic keratosis, squamous cell carcinoma (SCC), Bowen’s disease, basal cell carcinoma, and rarely, malignant adnexal tumors [3], sebaceous cell carcinoma, and sweat apparatus carcinoma [4,5]. Malignant transformations are primarily associated with HPV types 5 and 8 [6]. For increased survival, strict sun protection and lifetime monitoring for early detection of malignant lesions are essential.

## Case presentation

Case 1

A 42-year-old female with no known comorbidities, born of a non-consanguineous marriage, presented with an infected, non-healing ulcer in the right inguinal region for five months. She also noticed asymptomatic PV-like lesions on her body since the age of 18 years. The lesions started on her chest and then gradually progressed to involve other parts of her body. There were no similar complaints from her family members. The patient had a history of SCC of the vulva, for which excision and a split-skin graft were performed three years ago. The patient was advised to undergo radiotherapy, but she was not willing at that time.

**Figure 1:**
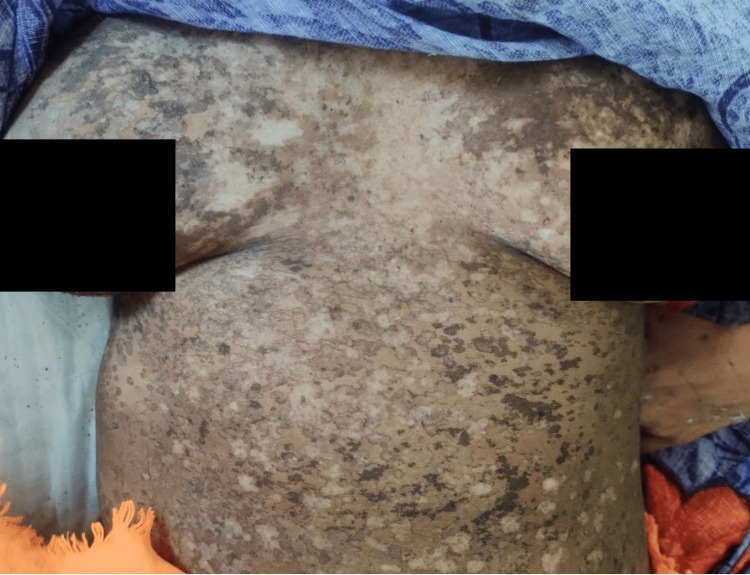
Clinical photo showing multiple well defined PV-like lesions on the trunk PV, pityriasis versicolor

**Figure 2:**
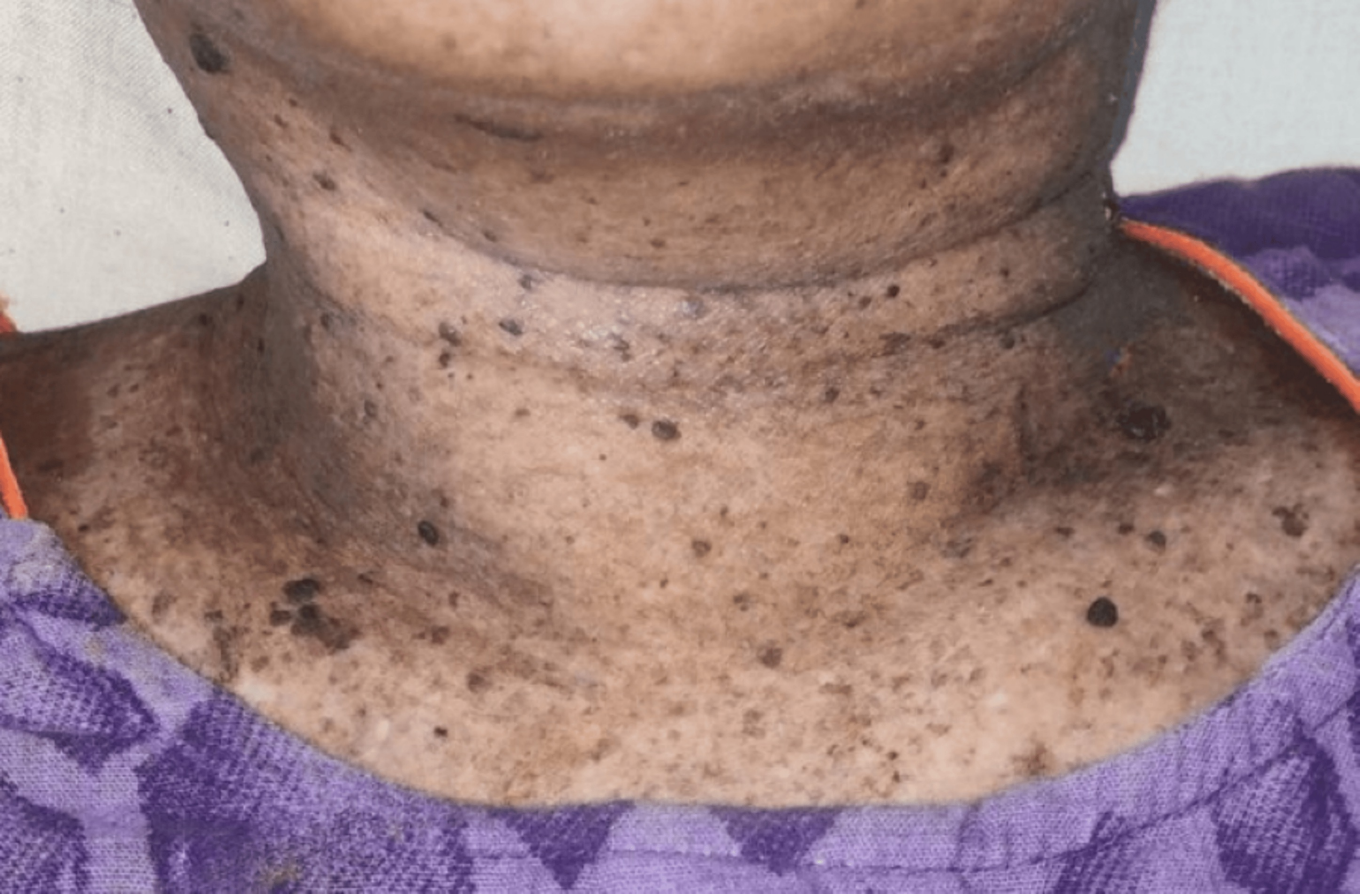
Clinical photo showing wart-like lesions on the neck

**Figure 3:**
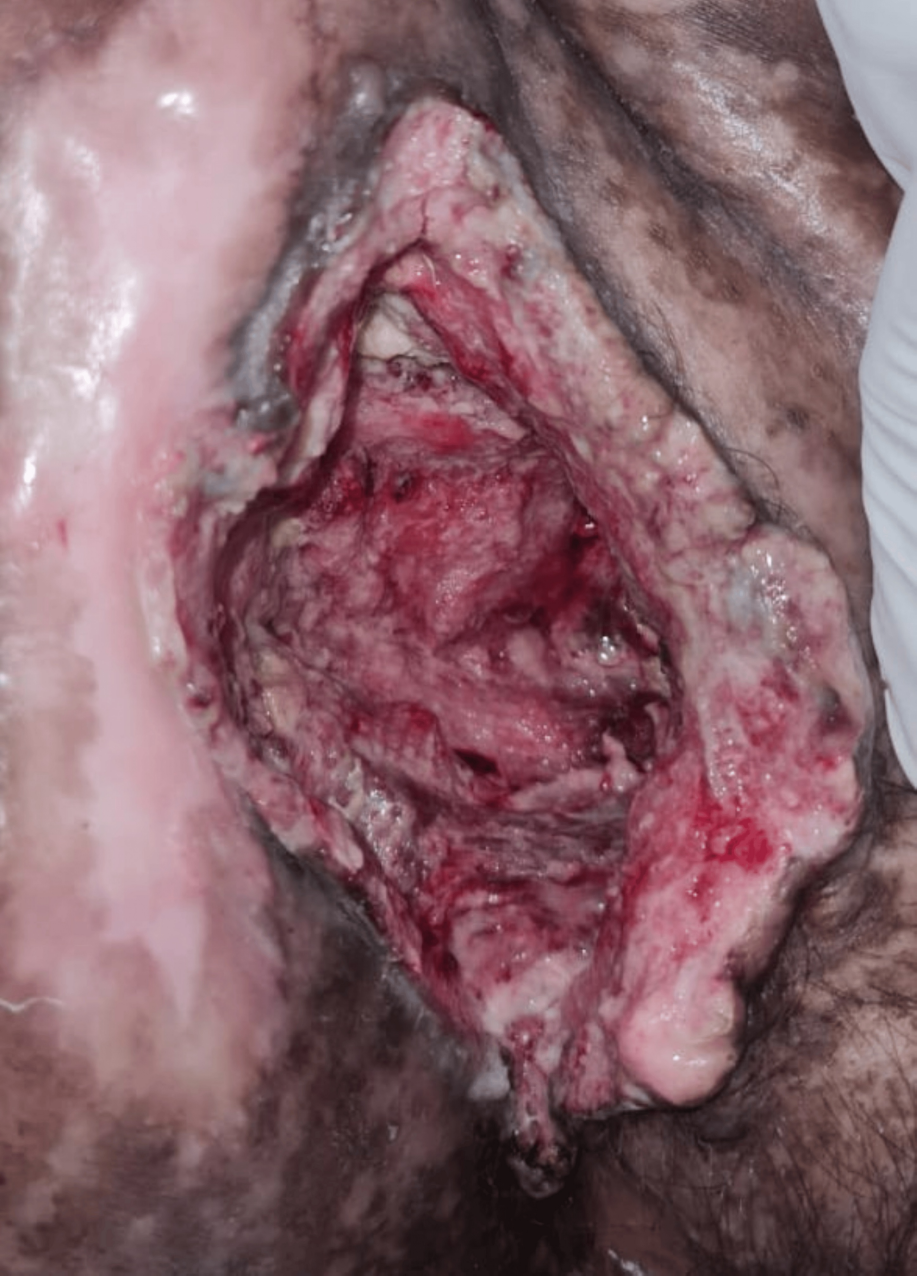
Clinical photo showing a large, single, non-healing, tender ulcer, with rolled-out edges in the right inguinal fossa

**Figure 4:**
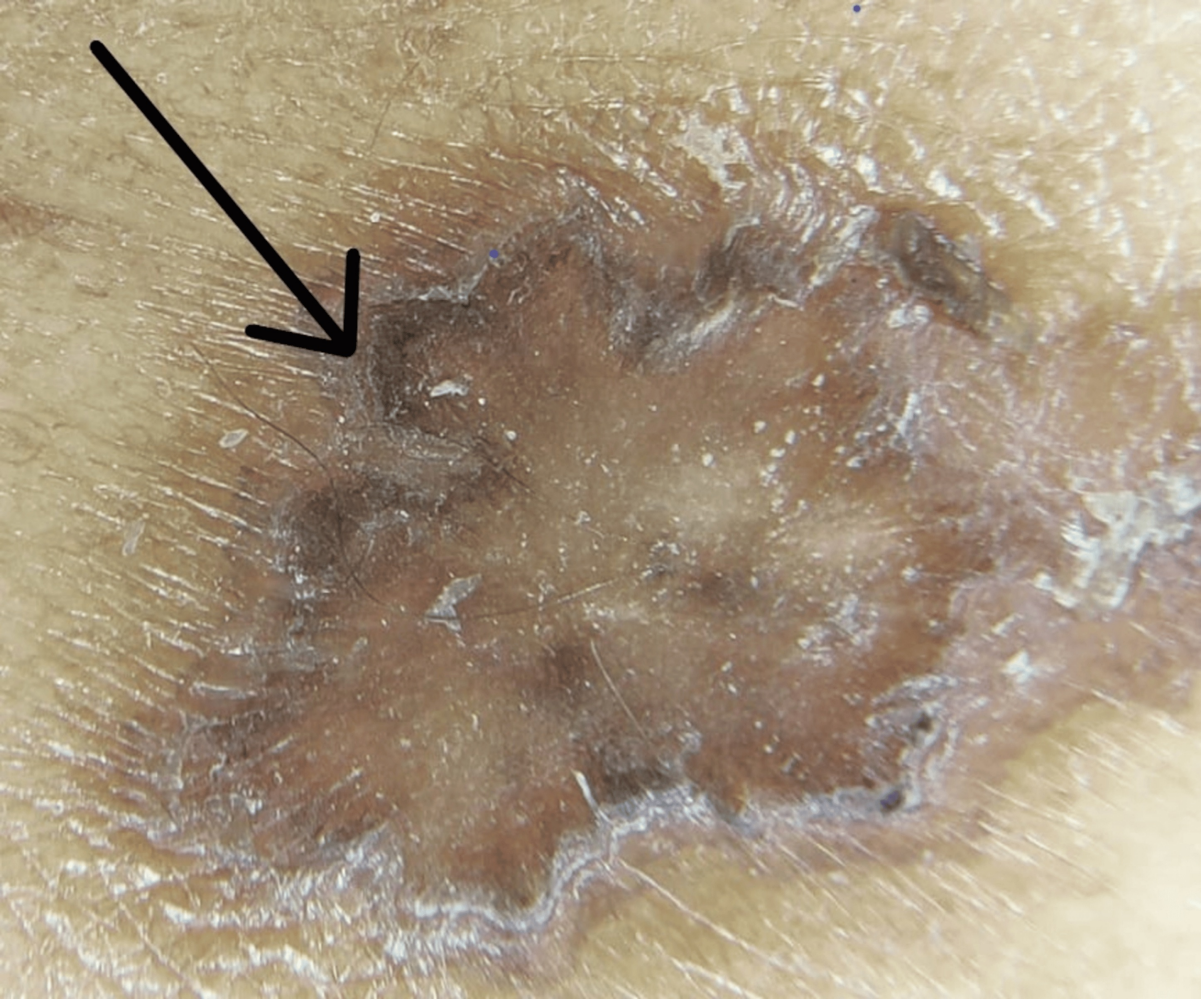
Dermascopic photo showing continuous marginal scaling

**Figure 5:**
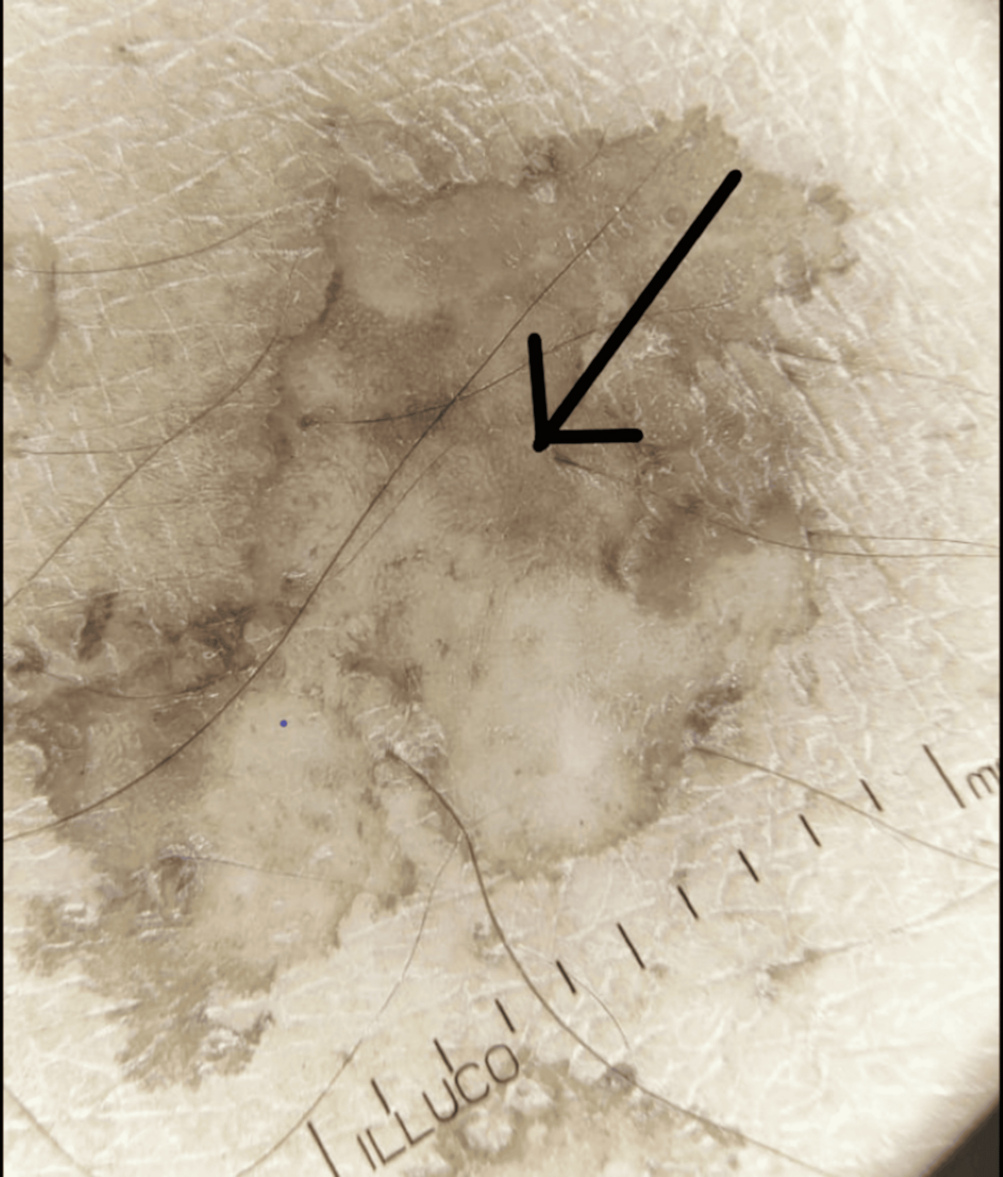
Dermascopic photo showing brownish-black areas mixed with hypopigmented area

On physical examination, multiple well-defined PV-like lesions were present on bilateral extremities and trunk (Figure 1). A few lesions had elevated borders. Multiple warty, keratotic papules were present on the face, neck, and chest (Figure 2). A large, single, non-healing, tender ulcer with pus, rolled-out edges, and a bony base was present in the right inguinal fossa, measuring 7 x 5 x 4 cm, with surrounding erythema (Figure 3). Active bleeding was observed when touching the base of the ulcer. Dermoscopy of a hyperpigmented macule was done, which showed continuous marginal scaling and brownish-black areas mixed with hypopigmented areas (Figures 4-5). 

**Figure 6:**
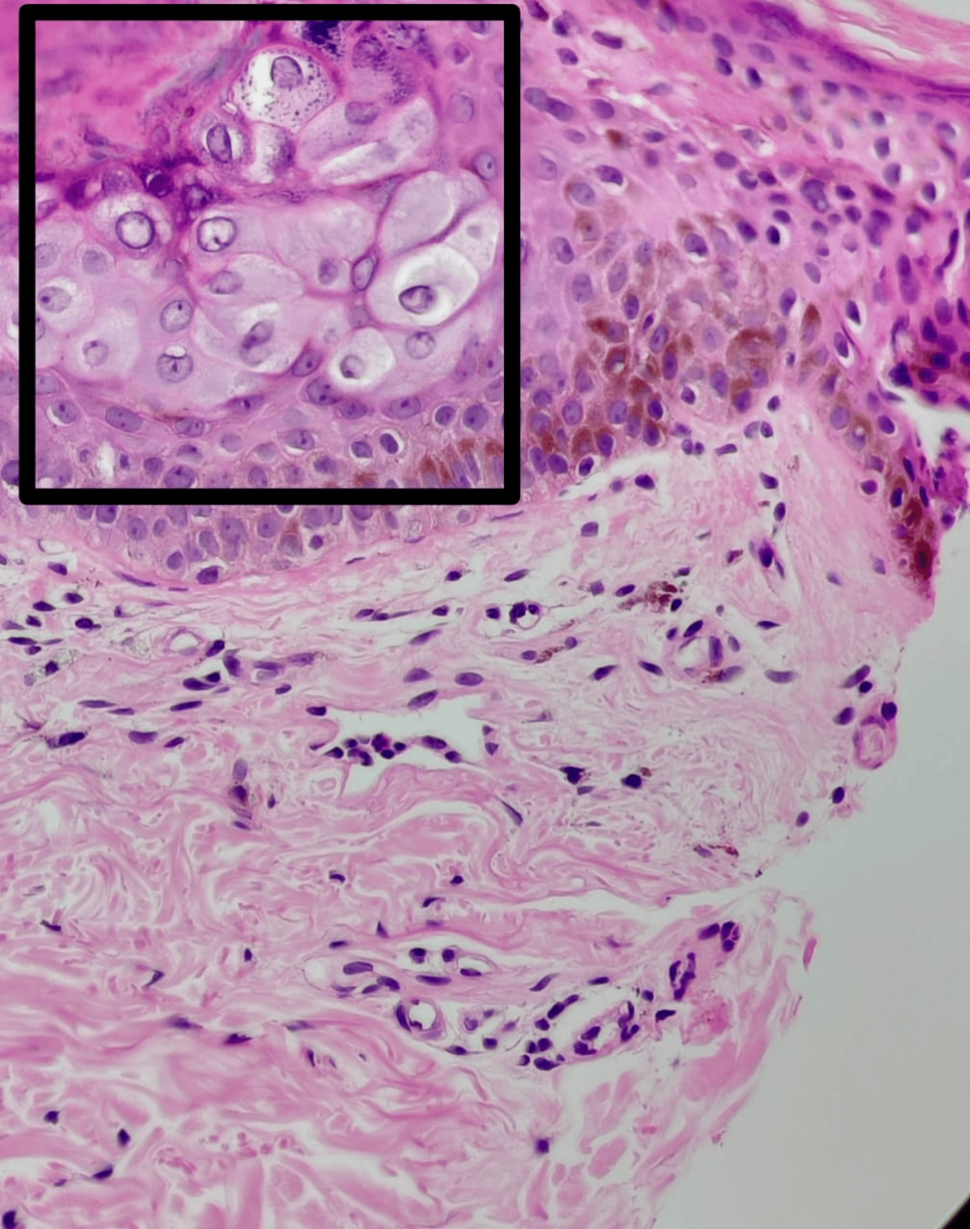
H&E stain of skin biopsy from the right arm under 400x magnification, showing epidermis with few keratinocytes with steel-grey cytoplasm, nuclear inclusions, and prominent basophilic granules H&E, hematoxylin and eosin

**Figure 7:**
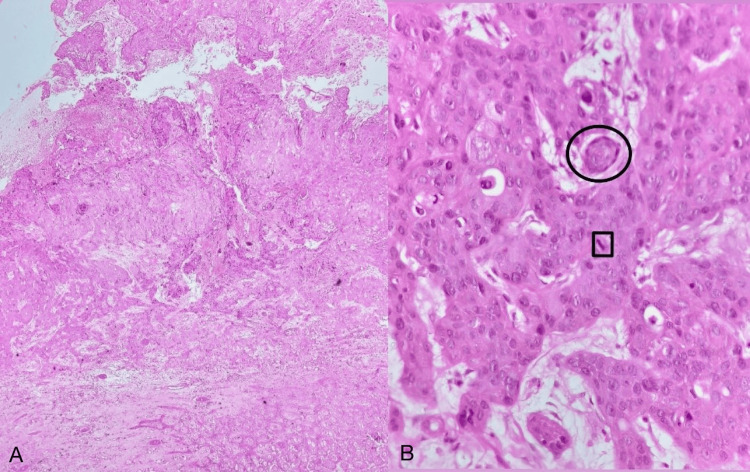
H&E staining of skin biopsy from the ulcer Figure (A) (40x magnification) and Figure (B) (400x magnification) show skin with focal ulceration and underlying subepithelium showing infiltration nests of moderately pleomorphic cells. The individual cells are large, polygonal, with indistinct cell borders, abundant eosinophilic cytoplasm, and round to oval vesicular nuclei with prominent nucleoli. Numerous atypical mitotic figures (black square) are noted. Individual cell keratinization and keratin pearl formation (black circle) are also seen. H&E, hematoxylin and eosin

On investigating, her total white blood cell count was elevated, and a pus culture from the ulcer in the right inguinal fossa showed *Escherichia coli* and *Pseudomonas aeruginosa*. Intravenous antibiotics were started according to the sensitivity report. The blood culture result was negative. A clinical diagnosis of EV with SCC was considered. The patient was started on intravenous antibiotics and other supportive measures. A skin punch biopsy was taken from a hyperpigmented lesion on the right arm, which showed epidermis with irregular acanthosis and marked hyperkeratosis with confluent parakeratosis. The upper epidermis showed a few keratinocytes with steel-grey cytoplasm, nuclear inclusions, and prominent basophilic granules. A superficial perivascular lymphocytic infiltrate was seen around the adnexa and blood vessels. Features suggestive of EV (Figure 6) and a biopsy from the non-healing ulcer were suggestive of SCC (Figures 7A-7B). Positron emission tomography-computed tomography (PET-CT) was done, which revealed thermal uptake in the right inguinal region, measuring 7.5 x 7.7 x 6.7 cm. The lesion was seen infiltrating the pectineus, obturator internus, obturator externus, and sartorius muscles on the right side. The lesion also caused erosion of the right femoral head, the anterior column of the acetabulum, and the right pubic bone. The right external iliac artery and vein were infiltrated by the mass. An oncologist was consulted, and radiotherapy was planned, but the patient passed away due to cardiac arrest.

Case 2

**Figure 8:**
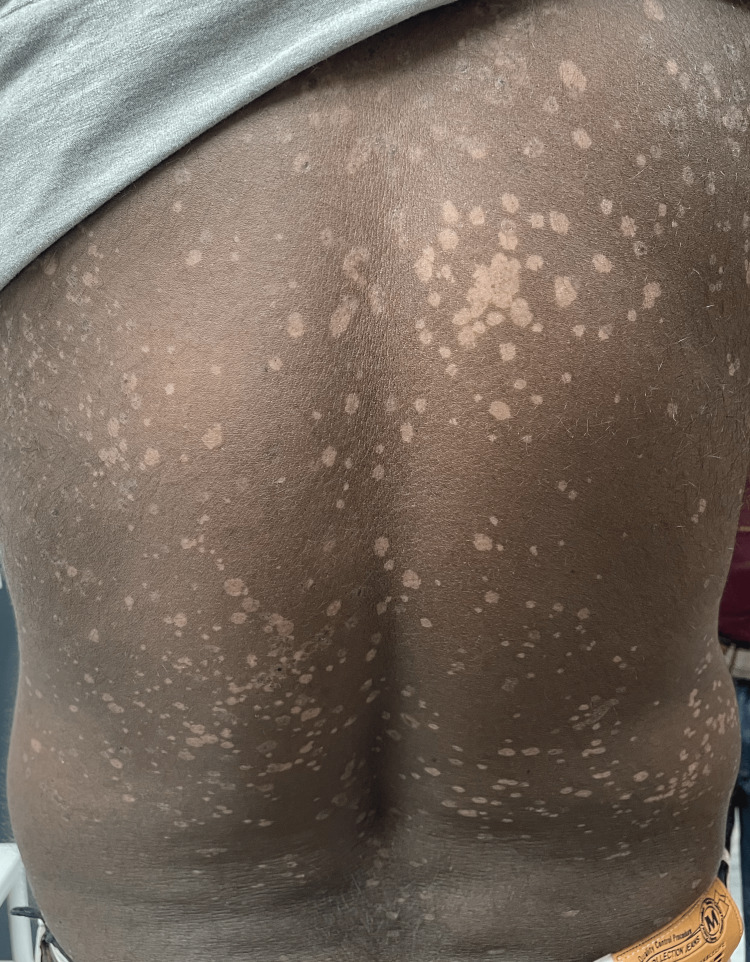
Clinical photo showing multiple well defined PV-like lesions on the trunk PV, pityriasis versicolor

**Figure 9:**
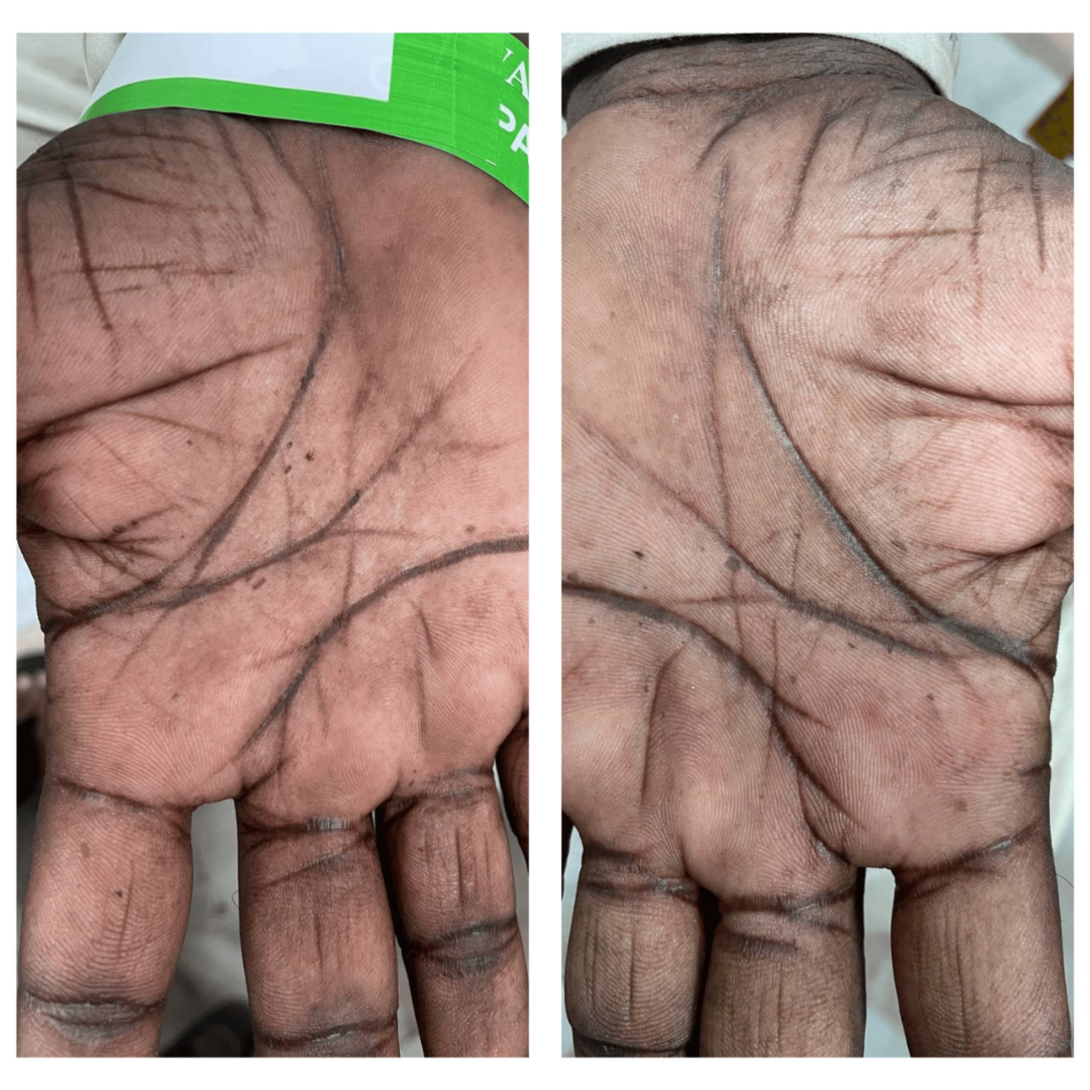
Clinical picture showing palmar pits in an EV patient EV, epidermodysplasia verruciformis

A 36-year-old male patient with no comorbidities, born of a non-consanguineous marriage, presented with complaints of multiple light and dark-colored lesions all over his body, which he had noticed 18 years ago. The lesions were insidious in onset and gradually progressive. He was otherwise normal and had no family history of similar complaints. On examination, multiple hypo- and hyperpigmented macules (Figure 8), along with a few plane wart-like lesions, were noted on the trunk, back, and bilateral upper and lower limbs. Bilateral palmar pits were noted (Figure 9). Hyperpigmentation was present in the oral cavity and buccal mucosa. A clinical diagnosis of EV was considered, and a skin biopsy was taken from the back, which showed epidermis with irregular acanthosis, enlarged keratinocytes with basophilic cytoplasm, and coarse blue granules. A perivascular halo was seen in keratinocytes. Superficial and deep perivascular lymphocytic infiltrates were seen. Features were consistent with EV. 

Baseline fasting lipid profile and liver function tests were done and were normal, after which the patient was started on oral retinoid (0.5 mg/kg/day).

Case 3

**Figure 10:**
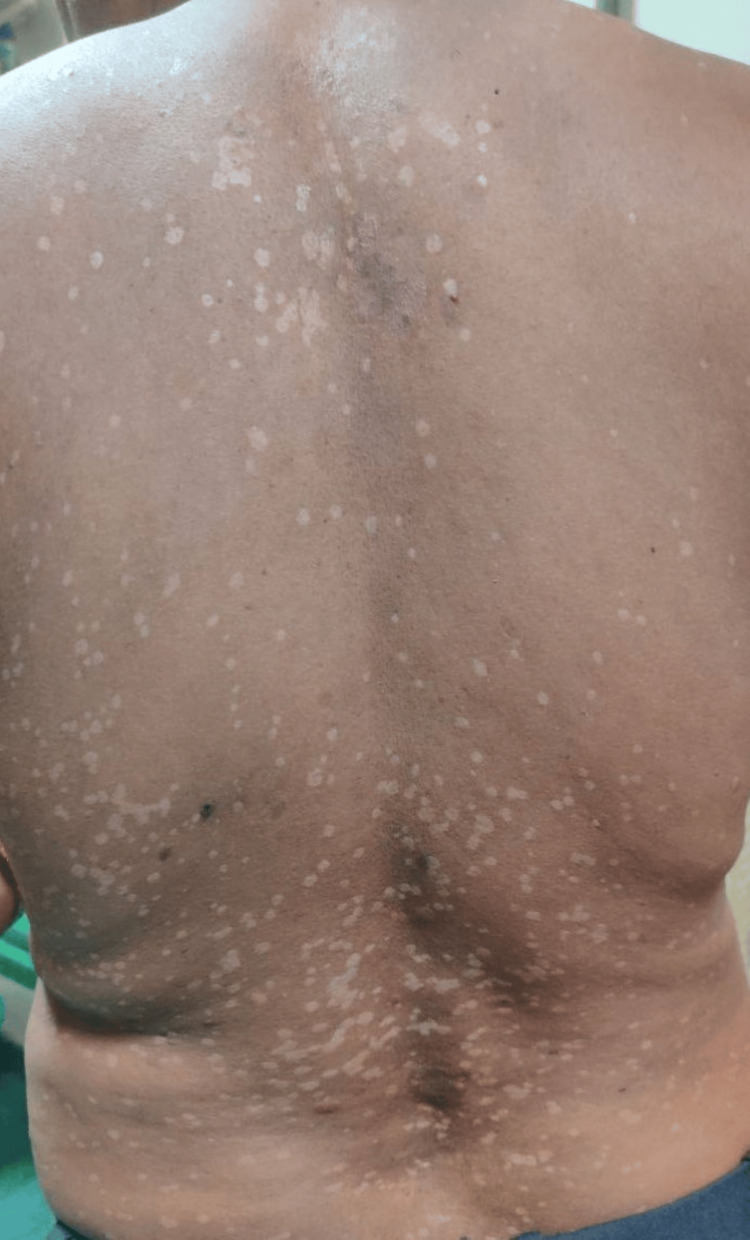
Clinical photo showing multiple well defined PV-like lesions on the trunk PV, pityriasis versicolor

A 44-year-old male patient with no comorbidities, born of a non-consanguineous marriage, presented with extensive PV-like and flat wart-like lesions over the trunk, back, and bilateral extremities (Figure 10), which he initially noticed 30 years ago. He was otherwise normal and had no family history of similar complaints. A clinical diagnosis of EV was made, and a 4-mm skin punch biopsy was taken from the back, which showed epidermis with regular acanthosis and focal broadening of rete ridges. The upper epidermis showed prominent granules with nuclei showing inclusions. The granular layer showed prominent koilocytes with raisinoid nuclei. Hyperkeratosis with mounds of parakeratosis was noted. Mild keratinocytic disarray and loss of nuclear polarity were noted in the epidermis. Features suggestive of EV were observed. Baseline fasting lipid profile and liver function tests were done and were normal, after which the patient was started on oral retinoids (0.5 mg/kg/day).

## Discussion

EV, also known as Lewandowski and Lutz dysplasia, is a rare autosomal recessive condition characterized by defective cell-mediated immunity and increased susceptibility to HPV, clinically presenting as progressive PV-like and wart-like lesions, which usually persist [2]. It does not exhibit any preference for a specific sex, race, or geographic location [7,8]. The illness is usually sporadic, but there are several reports of familial cases as well. To our knowledge, only two instances of autosomal dominant inheritance have also been documented so far. Apart from the mutations in the TMC6 and TMC8 genes in classic EV, mutations in CIB1 and variants in other genes involved in T cell and natural killer cell immunodeficiency, such as RHOH, CORO1A, CARMIL2, RASGRP1, MST1, DCLRE1C, DOCK8, and TTC7A, have also been reported [9]. The acquired type of EV is seen in immunocompromised or immunosuppressed individuals, with skin lesions presenting much later in life. More than 30 EV-associated HPV subtypes have been recognized, particularly a subset of beta HPV genotypes, like HPV 5, HPV 8, HPV 12, HPV 14, HPV 15, HPV 17, HPV 19, HPV 25, HPV 36, HPV 38, HPV 47, and HPV 50 [2], with HPV 5 and HPV 8 being the most common [10].

Here, we report three cases of EV with different spectra of disease. One patient, who had a long-standing case of EV, developed SCC over the vulva and inguinal region. Over their lifespan, SCC is known to develop in between 30% and 70% of patients with EV, and these lesions typically occur more commonly in sun-exposed areas [8]. In contrast, our patient developed SCC in a covered region, which is rare. In our literature search, only three such cases of SCC in covered areas, involving the scrotum and perianal region, were reported in patients with EV [11-13]. In the literature, there was a report of four patients who had synchronous EV and squamous high-grade intraepithelial lesion (HSIL) of the vulva, of which two patients had acquired EV and one patient had inherited EV [14]. In the second patient, in addition to classic skin lesions, he also had bilateral palmar pits, which is a rare association in EV. To our knowledge, only two case reports of EV patients with palmar pits have been published so far [15]. The third patient had classical cutaneous findings of EV. However, due to financial constraints, HPV typing was not done in our patients. It is important to elucidate the histopathological spectrum of EV and to differentiate it from its mimics, especially acrokeratosis verruciformis of Hopf [16].

Presently, there is no specific and effective therapy for this condition. Therefore, the goal of EV management is to stop benign lesions from becoming malignant by implementing preventive strategies such as photoprotection, genetic counseling, and early diagnosis of premalignant and malignant lesions [6]. For increased survival, strict sun protection and lifetime monitoring for early detection of malignant lesions are essential. Treatment options available for EV include topical retinoids, imiquimod, vitamin D analogs, oral retinoids, interferon, intralesional interferon therapy, cryotherapy, and immunotherapy. Techniques attempted for EV include cryotherapy, immunotherapy, interferons, electrodissection, photodynamic therapy, and Mohs micrographic surgery. The ideal medication for palliative care is acitretin (0.5-1 mg/day). Bowen's disease can be effectively treated with topical imiquimod applied five times a week. Photodynamic therapy with a 20% aminolevulinic acid (ALA) solution, using a red light emitting diode (LED) light source of 72 J/sq cm for 20 minutes weekly for three weeks, has shown no recurrence for five years [17]. Mohs micrographic surgery has been performed to treat widespread EV warts. If numerous vivid presentations of HPV infections occur, systemic interferon therapy (6 million units, weekly, twice) may be added.

## Conclusions

EV remains a complex and rare genetic condition that poses significant clinical challenges due to its strong association with HPV and the risk of developing SCC. The cases discussed in this report illustrate the variability in disease presentation and progression, including rare occurrences such as carcinoma in non-sun-exposed regions and the unusual presence of bilateral palmar pits. These findings highlight the necessity for personalized and vigilant patient management, emphasizing the importance of regular monitoring and early intervention to prevent malignant transformation. Moving forward, a multidisciplinary approach that includes genetic counseling, patient education, and rigorous photoprotection will be essential in managing EV and improving patient outcomes.
